# Participation in cancer rehabilitation and unmet needs: a population-based cohort study

**DOI:** 10.1007/s00520-012-1420-0

**Published:** 2012-03-14

**Authors:** Lise Vilstrup Holm, Dorte Gilså Hansen, Christoffer Johansen, Peter Vedsted, Pia Veldt Larsen, Jakob Kragstrup, Jens Søndergaard

**Affiliations:** 1 National Research Centre for Cancer Rehabilitation, Research Unit of General Practice, University of Southern Denmark, JB Winsløws vej 9A, 5000 Odense C, Denmark; 2 Danish Cancer Society Research Center, Strandboulevarden 49, 2100 Copenhagen, Denmark; 3 Research Unit for General Practice, University of Aarhus, Vennelyst Boulevard 6, 8000 Aarhus C, Denmark

**Keywords:** Cancer, Patient, Rehabilitation, Unmet needs, Participation

## Abstract

**Purpose:**

To investigate associations between cancer survivors’ sex, age, and diagnosis in relation to their (1) need for rehabilitation, (2) participation in rehabilitation activities, and (3) unmet needs for rehabilitation in a 14-month period following date of diagnosis.

**Methods:**

A population-based cohort study was performed on incident cancer patients diagnosed from 1 October 2007 to 30 September 2008. Fourteen months after diagnosis, participants completed a questionnaire developed to measure the aspects of rehabilitation. Logistic regression analyses were used to explore the association between sex, age, and diagnosis, and the outcome variables for rehabilitation.

**Results:**

A total of 3,439 patients participated, yielding an overall response rate of 70%. One third of the cancer patients reported a need for physical rehabilitation and one third for psychological rehabilitation. Half of the patients participated in at least one activity. Unmet needs were most often reported in psychological, sexual, and financial areas. Women expressed more needs, participated more often in rehabilitation activities, and had, to a higher extent, their emotional needs fulfilled. Breast cancer patients participated more often in physical rehabilitation. Elderly who expressed rehabilitation needs more often had them unresolved.

**Conclusions:**

A substantial variation in rehabilitation needs, participation in activities, and unmet needs in relation to sex, age, and cancer type was observed. Cancer care ought to systematically address the wide range of needs in all groups through integration of systematic needs assessment and targeted supply of offers.

## Introduction

Cancer survivors experience physical, psychological, work-related, and financial challenges and are potentially in need of individual and targeted rehabilitation [1, 2]. The World Health Organization (WHO) has defined rehabilitation as: “a process intended to enable people with disabilities to reach and maintain optimal physical, sensory, intellectual, psychological and social function” [3]. Hence, rehabilitation is wide-ranging and may encompass physical, psychological, work-related interventions, and financial support.

There is little knowledge about the overall number of cancer patients in need of rehabilitation efforts at different time points in the cancer trajectory. A Dutch cross-sectional study of patients with breast and bowel cancer (*n* = 147) found that 26% of the patients indicated a need for rehabilitation [4], while a cross-sectional study from Norway including the ten most frequent cancer types (*n* = 1,325) observed that 63% of the cancer patients reported a need for at least one rehabilitation service [5]. Based on nationwide and population-based register data, it has been estimated that up to 70% of all Danish cancer patients diagnosed within 1 year may have a need of rehabilitation of some kind [6]. Differences in the estimations of rehabilitation needs most likely reflect that most studies include different cancer types, obtain information at different points in time following diagnosis, and finally, define rehabilitation in various ways.

Besides needs, aspects of rehabilitation can also be illustrated through participation in rehabilitation and unmet needs. Two studies including only one or a few types of cancers (mainly breast cancer; *n* = 132 resp. *n* = 731) have assessed cancer patients’ actual utilization of rehabilitation activities, and typically, a third of the patients participated in an activity [7, 8]. The unmet needs for rehabilitation among cancer patients seem to be pronounced with regard to a variety of physical, psychological, and sexual problems [9, 10]. In smaller surveys among breast cancer patients, the problems have been shown to persist beyond the treatment phase [11, 12], and generally, most of the evidence in this area is derived from small studies including only patients with one type of cancer and most often, breast cancer [13–16].

It is reasonable to hypothesize that there is a substantial variation in cancer patients’ needs for and participation in rehabilitation, which, to some degree, can be explained by patient- and disease-related factors. Therefore, the aim of this study was to investigate associations between cancer survivors’ sex, age, and diagnosis in relation to their (1) need for rehabilitation, (2) participation in rehabilitation activities, and (3) unmet needs for rehabilitation in a 14-month period following date of diagnosis.

## Materials and methods

### Design

We performed a population-based cohort study including incident cancer patients, except patients with non-melanoma skin cancer, diagnosed from 1 October 2007 to 30 September 2008 in the Regions of Southern and Central Denmark (2.4 million residents) by obtaining information from hospital-based and national administrative registers. Information about rehabilitation issues was obtained from a patient questionnaire administered 14 months after diagnosis.

### Setting

The Danish health care system is primarily a publicly funded system [17]. More than 98% of the Danish population is listed with a general practitioner (GP), who acts as a gatekeeper to the rest of the health care system [18]. Since 2007, the 98 municipalities have had the responsibility for most rehabilitation of patients with chronic disease, including cancer, while hospitals are still responsible for highly specialized rehabilitation [19]. The activities may include physiotherapy, other physical training, counseling with psychologist, dietary advice, counseling with social worker, occupational therapy, patient education, and smoking cessation counseling, but the offers vary across the country. Like other health services, rehabilitation provided by the public healthcare system and the municipalities is free of charge.

Cancer patients may participate in rehabilitation activities outside the public healthcare system and the municipalities. The Danish Cancer Society is a private patient organization offering patient support and counseling free of charge at counseling centers all over the country [20]. Furthermore, patients may seek self-financed rehabilitation from, e.g., private physiotherapists, psychologists, and alternative practitioners.

### Study population

The study population was defined by the following means:

All residents of Denmark are assigned a unique ten-digit personal identification number, which includes information on date of birth and sex. This Personal Identification Number (CPR) is the key variable in linkages between public, health, and disease registries in the country [21].

From the regional hospitals’ Patient Administrative System [22], we obtained information on all patients diagnosed with cancer (ICD 10 codes DC00-96, DD37-48) during the study period. The cancers were grouped into breast cancer, prostate, colo-rectal, gynecological, malignant melanoma, lung, lymphoma, head and neck, and other cancers. Furthermore, the cancers should be given an additional code, “the AZCA-1 code,” which was the code for the first time the department had an encounter with the patient regarding the cancer. We included only patients aged 18 years or older listed with a GP in the two regions. Based on the National Patient Registry [23], we identified all patients in our sample with a previous diagnosis of cancer to ensure that the study population comprised incident cancer patients only.

Following identification by the administrative sampling procedure, each patient’s GP was mailed a questionnaire to confirm that a cancer was diagnosed.

This cohort of incident cancer patients was established for the use in several research projects, and 6 months following date of diagnosis, patients were mailed a questionnaire, which included a request for them to confirm that they had cancer for the first time and giving them the possibility of declining the use of the information given by their GP. Prior to the distribution of the 14-month patient questionnaires, vital status and postal address were confirmed by linkage to the Civil Registration System [21], which is continuously updated on these matters. Non-responders were sent a reminder after 3 weeks. All letters included the questionnaire and a prepaid return envelope.

### Development of the 14-month patient questionnaire

The patient questionnaire comprised 171 items and was designed to give information about various aspects of cancer rehabilitation [24], including those presented in this study. Ad hoc questions covering the three different aspects of rehabilitation (1) perceived needs, (2) participation, and (3) unmet needs were developed with an empirical background established through extensive literature review, as well as a report and a PhD thesis on the subject [25, 26], keeping WHO’s definition of rehabilitation in mind. The list of rehabilitation activities was guided by activities present in the municipalities, as well as activities provided to residents by the Danish Cancer Society.

The questionnaire was pilot-tested and revised in a three-step procedure. Researchers active in the field of cancer rehabilitation were asked to comment on content, layout, volume, and intelligibility of the draft. Subsequently, ten cancer patients were asked to fill in the questionnaire, and in a semi-structured interview with the first author, these patients provided comments on content, layout, volume, and intelligibility. Based on all these pilot activities, questions were revised and a new draft was completed. The last pilot study included 100 cancer patients, who were asked to fill in a mailed version, enabling us to examine discrimination and acceptability and make the final corrections. The overall participation rate in this pilot study was 75%.

### Data on rehabilitation

The rehabilitation variables covered self-assessed perceived need for rehabilitation, participation in rehabilitation activities, and unmet rehabilitation needs from time of cancer diagnosis until the day of filling in the questionnaire.

“Need for rehabilitation during the 14-month period” and “unmet rehabilitation after 14 months” were asked for thematically, i.e., “physical,” “emotional,” “family oriented,” “sexual,” “work-related,” and “financial” areas.

As an example, the following questions were asked with regard to physical needs:

1. To what extent from diagnosis and until now have you needed professional help with physical problems? (response: “not at all,” “to a small extent,” “to some extent,” and “to a great extent”)
2. Until now, to what extent have you had your needs fulfilled in terms of help with physical problems? (response: “not at all,” “to a small extent,” “to some extent,” “to a great extent,” and “not relevant”)

Similar questions were asked for all areas. Patients were categorized as having an “unmet need” if they had expressed a “need for rehabilitation” during the 14-month period, and the need, to some extent, was not fulfilled after 14 months.

“Participation in rehabilitation activities” was assessed by asking:

3. Have you from diagnosis and until now participated in any of the following activities due to problems caused by your cancer disease? (listing of possible providers/activities)

Three categories of activities were defined based on profession of the provider/activity: (1) “Physical activities” (physiotherapist, occupational therapist, chiropractor, patient education, smoking cessation counseling, nutritional information, physical training, and alternative practitioner including acupuncturist and reflexologist), (2) “Psychological activities” (psychologist, marriage counselor or sexologist, supportive group sessions or patient associations, and spiritual counseling), and (3) “work-related/financial activities” (social worker, union representative or employer, financial or insurance counselor). Furthermore, the variable “Participation in one or more activities” was defined based on the above-mentioned categories.

### Statistical analysis

Need for rehabilitation was dichotomized into “no need” (“not at all”) and “need” (combining “to a small extent,” “to some extent”, and “to a great extent”), and similarly, unmet need was dichotomized into “unmet need” and “met need.” Answers in the “not relevant” category were excluded from the analyses. For analyses regarding “unmet rehabilitation needs after 14 months,” only patients expressing a “need for rehabilitation” were included.

Univariate and multiple logistic regression analyses were used to explore the association between sex, age, and diagnosis, and the rehabilitation variables. In presence of an interaction between age group and sex, the analyses were stratified on sex. All tests were two-sided, and *p* < 0.05 was considered statistically significant. Adjusted odds ratios (OR_adj_) are presented with 95% confidence intervals (95% CI). Analyses were performed using Stata Release 11 (StataCorp, College Station, TX, USA).

## Results

**Fig. 1:**
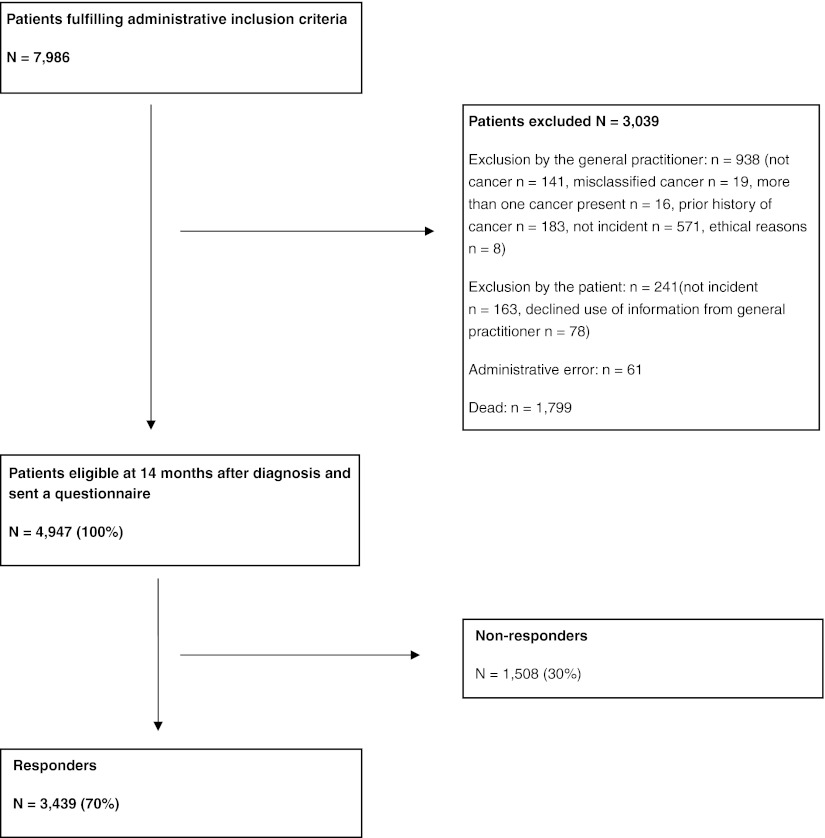
Inclusion of patients into the study

Of the 4,947 subjects eligible at 14 months, 3,439 returned the questionnaire (70%) (Fig. 1). Table 1 shows responders and non-responders with regard to sex, age, and diagnoses.

**Table 1:** Medical and demographic characteristics of responders and non-responders in a cohort study of cancer rehabilitation

	Responders, *n* (%)	Non-responders, *n* (%)
	3,439 (69.5)	1,508 (30.5)
Sex		
Men	1,466 (42.6)	712 (47.2)
Women	1,973 (57.4)	796 (52.8)
Age (years)		
18–39	152 (4.4)	118 (7.8)
40–49	331 (9.6)	154 (10.2)
50–59	660 (19.2)	292 (19.4)
60–69	1,263 (36.8)	419 (27.8)
70–79	802 (23.3)	338 (22.4)
80+	231 (6.7)	187 (12.4)
Cancer diagnoses		
Breast	976 (28.4)	314 (20.8)
Prostate	501 (14.6)	179 (11.9)
Colo-rectal	522 (15.2)	213 (14.1)
Gynecological	230 (6.7)	120 (7.9)
Malignant melanoma	233 (6.8)	102 (6.8)
Lung	188 (5.5)	113 (7.5)
Lymphoma	104 (3.0)	44 (2.9)
Head and neck	125 (3.6)	81 (5.4)
Other	560 (16.2)	342 (22.7)

### Need for rehabilitation during the 14-month period

Self-perceived need for physical and psychological rehabilitation was equally frequent, 32% and 31%, respectively (Table 2). Overall, the higher the age, the less likely the patients were to express a need for rehabilitation. Women expressed rehabilitation needs in emotional, physical, family oriented, and work-related areas more often than men.

**Table 2:** Needs for rehabilitation during 14 months following time of diagnosis

	Physical area			Emotional area			Family oriented area			Sexual area				Work-related area			Financial area		
	*n* = 3,242			*n* = 3,254			*n* = 3,250			*n* = 3,197				*n* = 1,276			*n* = 1,895		
	*n* (%)	ORcrude	ORadj^a^ (95% CI)	*n* (%)	ORcrude	ORadj^a^ (95% CI)	*n* (%)	ORcrude	ORadj^a^ (95% CI)	*n* (%)	ORcrude	Men *n* = 1,366 ORadj^b^ (95% CI)	Women *n* = 1,829 ORadj^b^ (95% CI)	*n* (%)	ORcrude	ORadj^a^ (95% CI)	*n* (%)	ORcrude	ORadj^a^ (95% CI)
Needs, total	1,028 (31.7)	−	−	997 (30.6)	−	−	453 (13.9)	−	−	529 (16.6)	−	−	−	252 (19.8)	−	−	257 (13.4)	−	−
Sex																			
Men	352 (25.4)	1.00	1.00	327 (23.5)	1.00	1.00	145 (10.4)	1.00	1.00	308 (22.5)	1.00	−	−	77 (14.3)	1.00	1.00	118 (13.8)	1.00	1.00
Women	676 (36.5)	1.69**	1.36** (1.01–1.70)	670 (35.9)	1.82**	1.72** (1.37–2.17)	308 (16.6)	1.70**	1.40* (1.04–1.88)	221 (12.1)	0.47**	−	−	175 (23.7)	1.87**	1.55* (1.01–2.39)	139 (13.4)	0.97	0.86 (0.60–1.25)
Age (years)																			
18–39	54 (36.0)	1.00	1.00	82 (54.3)	1.00	1.00	47 (31.3)	1.00	1.00	34 (22.5)	1.00	1.00	1.00	23 (22.3)	1.00	1.00	20 (16.7)	1.00	1.00
40–49	149 (46.4)	1.54*	1.35 (0.90–2.03)	176 (53.8)	0.98	0.86 (0.57–1.28)	96 (29.3)	0.91	0.82 (0.53–1.27)	77 (23.7)	1.07	0.83 (0.35–2.02)	0.91 (0.51–1.62)	73 (32.0)	1.64	1.43 (0.82–2.51)	51 (19.5)	1.21	1.33 (0.74–2.38)
50–59	263 (41.3)	1.25	1.13 (0.77–1.65)	279 (43.8)	0.66*	0.59** (0.40–0.85)	135 (21.2)	0.59**	0.55** (0.36–0.83)	133 (21.0)	0.91	0.93 (0.43–1.99)	0.61 (0.35–1.06)	116 (28.9)	1.41	1.27 (0.74–2.17)	97 (19.5)	1.21	1.31 (0.76–2.27)
60–69	339 (27.8)	0.68*	0.63* (0.43–0.91)	304 (25.0)	0.28**	0.25** (0.17–0.36)	117 (9.6)	0.23**	0.22** (0.14–0.33)	203 (17.0)	0.71	0.72 (0.34–1.51)	0.25** (0.14–0.44)	32 (9.8)	0.38**	0.37** (0.20–0.69)	66 (10.8)	0.61	0.65 (0.37–1.15)
70–79	172 (23.6)	0.55**	0.53** (0.36–0.78)	125 (17.0)	0.17**	0.16** (0.11–0.24)	48 (6.6)	0.15**	0.15** (0.09–0.24)	69 (9.7)	0.37**	0.33** (0.15–0.72)	0.04** (0.01–0.11)	6 (3.6)	0.13**	0.12** (0.05–0.32)	18 (5.6)	0.30**	0.30** (0.15–0.61)
80+	51 (27.4)	0.67	0.64 (0.39–1.03)	31 (16.4)	0.17**	0.15** (0.09–0.26)	10 (5.3)	0.12**	0.12** (0.06–0.25)	13 (7.1)	0.26**	0.26** (0.10–0.69)	0.06** (0.01–0.26)	2 (4.0)	0.14*	0.14* (0.03–0.63)	5 (6.0)	0.32*	0.35 (0.12–1.00)
Cancer diagnosis																			
Breast	367 (39.5)	1.00	1.00	338 (36.2)	1.00	1.00	152 (16.3)	1.00	1.00	111 (12.1)	1.00	−	1.00	94 (23.9)	1.00	1.00	62 (11.7)	1.00	1.00
Prostate	96 (20.7)	0.40**	0.70* (0.50–0.99)	89 (19.1)	0.42**	1.16 (0.80–1.66)	30 (6.4)	0.35**	0.85 (0.51–1.43)	159 (34.3)	3.79**	2.44** (1.67–3.56)	−	15 (9.7)	0.34**	1.11 (0.52–2.36)	22 (8.2)	0.67	0.93 (0.49–1.78)
Colo-rectal	142 (29.0)	0.62**	0.84 (0.65–1.10)	109 (22.2)	0.51*	0.88 (0.66–1.18)	50 (10.2)	0.58**	0.96 (0.66–1.41)	68 (14.2)	1.20	1.00	1.11 (0.65–1.89)	31 (16.8)	0.64	1.17 (0.69–1.98)	32 (11.7)	0.99	1.17 (0.70–1.96)
Gynecological	69 (31.9)	0.72*	0.72* (0.52–0.99)	79 (36.1)	0.99	1.00 (0.72–1.38)	43 (19.6)	1.25	1.25 (0.85–1.85)	41 (19.1)	1.71**	−	1.83** (1.21–2.77)	26 (30.2)	1.38	1.36 (0.80–2.32)	22 (17.3)	1.58	1.64 (0.96–2.82)
Malignant melanoma	49 (22.4)	0.44**	0.48** (0.33–0.68)	51 (23.2)	0.53**	0.52** (0.35–0.75)	19 (8.6)	0.49**	0.43* (0.25–0.73)	16 (7.3)	0.57*	0.34** (0.15–0.75)	0.34** (0.16–0.75)	8 (8.3)	0.29**	0.36* (0.16–0.80)	13 (10.0)	0.84	0.83 (0.43–1.62)
Lung	71 (39.7)	1.01	1.43* (1.01–2.04)	70 (39.3)	1.14	2.19** (1.52–3.16)	28 (15.6)	0.95	1.65* (1.03–2.66)	27 (15.7)	1.35	0.99 (0.53–1.84)	1.53 (0.74–3.16)	8 (15.7)	0.59	1.09 (0.47–2.54)	18 (17.1)	1.56	2.00* (1.07–3.75)
Lymphoma	35 (34.3)	0.80	0.92 (0.59–1.44)	49 (48.0)	1.63*	2.24 **(1.42–3.51)	24 (23.8)	1.60	1.90* (1.12–3.23)	15 (15.0)	1.28	0.87 (0.40–1.87)	0.76 (0.29–2.02)	9 (19.6)	0.78	0.93 (0.41–2.10)	11 (16.2)	1.46	1.39 (0.67–2.91)
Head and neck	32 (26.9)	0.56**	0.69 (0.43–1.09)	39 (33.3)	0.88	1.29 (0.82–2.04)	15 (12.9)	0.76	0.97 (0.52–1.80)	12 (10.4)	0.84	0.31** (0.13–0.72)	1.49 (0.54–4.11)	14 (25.5)	1.09	1.57 (0.74–3.32)	12 (15.0)	1.33	1.20 (0.58–2.50)
Other	167 (31.9)	0.72**	0.92 (0.70–1.20)	173 (32.9)	0.87	1.29 (0.98–1.70)	92 (17.6)	1.09	1.42* (1.01–2.02)	80 (15.4)	1.32	0.81 (0.52–1.25)	1.07 (0.64–1.78)	47 (22.5)	0.93	1.38 (0.84–2.28)	65 (20.8)	1.99	2.03** (1.28–3.22)

For each area (physical, emotional, family oriented, sexual, work-related, and financial) number, frequencies and crude and adjusted odds ratios (ORs) are shown with regard to sex, age, and cancer diagnosis

^a^Adjusted for sex, age group, and cancer diagnosis

^b^Adjusted for age group and cancer diagnosis

*p < 0.05

**p < 0.01

### Participation in rehabilitation activities

Overall, 52% had participated in at least one rehabilitation activity (Table 3). Physical activities were used by 42%, psychological by 17%, and work-related/finance-related activities by 12%. The single most used activity was physiotherapy (31%), followed by physical training (15%), psychologist (11%), dietician (10%), alternative practitioner (including acupuncturist or reflexologist) (7%), and social worker (6%) (data not shown). Women were more likely to participate in physical and psychological activities, while no sex difference was observed with regard to counseling about work/economy. The oldest patients were less likely to participate in activities. Patients with breast cancer participated more frequently in physical activities compared with other cancer patients.

**Table 3:** Participation in rehabilitation activities during 14 months following time of diagnosis

	One or more activities			One or more physical activities			One or more psychological activities			One or more work-related/financial activities		
	*n* = 3,257			*n* = 3,439			*n* = 3,439			*n* = 3,439		
	*n* (%)	ORcrude	ORadj^a^ (95% CI)	*n* (%)	ORcrude	ORadj^a^ (95% CI)	*n* (%)	ORcrude	ORadj^a^ (95% CI)	*n* (%)	ORcrude	ORadj^a^ (95% CI)
Participation, total	1,697 (52.1)	−	−	1,447 (42.1)	−	−	586 (17.0)	−	−	403 (11.7)	−	−
Sex												
Men	545 (39.4)	1.00	1.00	438 (29.9)	1.00	1.00	152 (10.4)	1.00	1.00	134 (9.1)	1.00	1.00
Women	1,152 (61.5)	2.46**	1.54** (1.25–1.90)	1009 (51.1)	2.46**	1.41** (1.14–1.73)	434 (22.0)	2.44**	1.90** (1.43–2.52)	269 (13.6)	1.56**	1.03 (0.74–1.43)
Age (years)												
18–39	102 (67.1)	1.00	1.00	68 (44.7)	1.00	1.00	63 (41.5)	1.00	1.00	55 (36.2)	1.00	1.00
40–49	234 (72.2)	1.27	0.88 (0.57–1.37)	197 (59.5)	1.82**	1.29 (0.85–1.96)	118 (35.6)	0.78	0.59* (0.39–0.89)	87 (26.3)	0.63*	0.50** (0.32–0.78)
50–59	443 (69.7)	1.13	0.81 (0.54–1.22)	344 (52.1)	1.34	1.02 (0.70–1.50)	184 (27.9)	0.55**	0.42** (0.29–0.63)	163 (24.7)	0.58**	0.48** (0.32–0.72)
60–69	600 (49.6)	0.48**	0.34** (0.23–0.51)	546 (43.2)	0.94	0.74 (0.51–1.08)	154 (12.2)	0.20**	0.16** (0.11–0.24)	71 (5.6)	0.11**	0.09** (0.06–0.13)
70–79	257 (34.7)	0.26**	0.21** (0.14–0.32)	238 (29.7)	0.52**	0.48** (0.33–0.71)	53 (6.6)	0.10**	0.09** (0.06–0.14)	19 (2.4)	0.04**	0.04** (0.02–0.07)
80+	61 (31.4)	0.22**	0.18** (0.11–0.29)	54 (23.4)	0.38**	0.34** (0.21–0.55)	14 (6.1)	0.10**	0.08** (0.04–0.15)	8 (3.5)	0.06**	0.05** (0.02–0.12)
Cancer diagnosis												
Breast	694 (73.9)	1.00	1.00	652 (66.8)	1.00	1.00	239 (24.5)	1.00	1.00	152 (15.6)	1.00	1.00
Prostate	163 (35.0)	0.19**	0.41** (0.30–0.57)	139 (27.7)	0.19**	0.34** (0.25–0.47)	34 (6.8)	0.22**	0.77 (0.47–1.24)	23 (4.6)	0.26**	0.65 (0.36–1.17)
Colo-rectal	192 (39.8)	0.23**	0.36** (0.27–0.47)	149 (28.5)	0.20**	0.28** (0.22–0.36)	70 (13.4)	0.48**	0.91 (0.65–1.27)	47 (9.0)	0.54**	0.84 (0.56–1.27)
Gynecological	107 (47.8)	0.32**	0.31** (0.23–0.42)	78 (33.9)	0.26**	0.25** (0.19–0.34)	47 (20.4)	0.79	0.76 (0.53–1.11)	30 (13.0)	0.81	0.77 (0.49–1.21)
Malignant melanoma	71 (32.3)	0.17**	0.16** (0.11–0.22)	52 (22.3)	0.14**	0.16** (0.11–0.22)	25 (10.7)	0.37**	0.32** (0.20–0.52)	12 (5.2)	0.29**	0.20** (0.10–0.38)
Lung	93 (52.0)	0.38**	0.62** (0.43–0.89)	75 (39.9)	0.33**	0.46** (0.33–0.65)	31 (16.5)	0.61*	1.22 (0.78–1.92)	15 (8.0)	0.47**	0.82 (0.44–1.50)
Lymphoma	54 (54.0)	0.41**	0.47** (0.30–0.74)	45 (43.3)	0.38**	0.44** (0.28–0.67)	25 (24.0)	0.98	1.24 (0.74–2.08)	30 (28.9)	2.20**	2.36** (1.39–4.03)
Head and neck	69 (58.5)	0.50*	0.65 (0.42–1.01)	54 (43.2)	0.38**	0.49** (0.32–0.74)	27 (21.6)	0.85	1.35 (0.81–2.25)	11 (8.8)	0.52*	0.49* (0.24–0.99)
Other	254 (48.0)	0.33**	0.44** (0.33–0.57)	203 (36.3)	0.28**	0.37** (0.29–0.48)	88 (15.7)	0.57**	0.83 (0.60–1.15)	83 (14.8)	0.94	1.01 (0.69–1.47)

For each area (one or more activities, one or more physical activities, one or more psychological activities, and one of more work-related/financial activities) number, frequencies and crude and adjusted odds ratios (ORs) are shown with regard to sex, age, and cancer diagnosis

^a^Adjusted for sex, age group, and cancer diagnosis

*p < 0.05

**p < 0.01

### Unmet rehabilitation needs after 14 months

Among patients who had expressed a need for rehabilitation during the 14-month period, unmet needs were most common for sexual problems (50%) and least common for physical problems (17%) (Table 4). Financial issues were still unsolved for one third. Men were more likely to have emotional unmet needs than women, and higher age was associated with a greater likelihood of unmet needs in all areas, except for physical and financial problems. Compared with patients with breast cancer, unmet needs for physical rehabilitation were more common among patients with colo-rectal, gynecological, and head and neck cancers.

**Table 4:** Unmet needs for rehabilitation 14 months after diagnosis

	Physical area			Emotional area			Family oriented area			Sexual area			Work-related area			Financial area		
	*n* = 922			*n* = 883			*n* = 380			*n* = 454			*n* = 222–230			*n* = 230		
	*n* (%)	ORcrude	ORadj^a^ (95% CI)	*n* (%)	ORcrude	ORadj^a^ (95% CI)	*n* (%)	ORcrude	ORadj^a^ (95% CI)	*n* (%)	ORcrude	ORadj^a^ (95% CI)	*n* (%)	ORcrude	ORadj^a^ (95% CI)	*n* (%)	ORcrude	ORadj^a^ (95% CI)
Unmet needs, total	159 (17.3)	−	−	209 (23.7)	−	−	122 (32.1)	−	−	225 (49.6)	−	−	43 (18.7)	−	−	79 (34.4)	−	−
Sex																		
Men	63 (19.8)	1.00	1.00	85 (31.1)	1.00	1.00	43 (34.4)	1.00	1.00	124 (46.8)	1.00	1.00	18 (26.1)	1.00	1.00	42 (39.3)	1.00	1.00
Women	96 (15.9)	0.76	0.96 (0.60–1.54)	124 (20.3)	0.56**	0.58** (0.37–0.92)	79 (31.0)	0.86	1.06 (0.58–1.93)	101 (53.4)	1.31	0.80 (0.42–1.55)	25 (15.5)	0.52	0.53 (0.19–1.47)	37 (30.1)	0.67	0.53 (0.24–1.19)
Age (years)																		
<60	64 (15.3)	1.00	1.00	91 (18.3)	1.00	1.00	65 (26.9)	1.00	1.00	105 (47.7)	1.00	1.00	33 (16.6)	1.00	1.00	53 (33.3)	1.00	1.00
≥60	95 (18.9)	1.29	1.17 (0.81–1.70)	118 (30.7)	1.98**	2.05** (1.47–2.85)	57 (41.3)	1.92**	2.03** (1.27–3.25)	120 (51.3)	1.15	1.83** (1.16–2.89)	10 (32.3)	2.40*	2.98* (1.14–7.80)	26 (36.6)	1.16	1.08 (0.58–2.03)
Cancer diagnosis																		
Breast	40 (12.1)	1.00	1.00	55 (17.7)	1.00	1.00	33 (26.4)	1.00	1.00	54 (56.8)	1.00	1.00	12 (14.1)	1.00	1.00	19 (38.0)	1.00	1.00
Prostate	16 (18.4)	1.63	1.46 (0.65–3.27)	24 (30.8)	2.06*	0.94 (0.45–1.97)	7 (28.0)	1.08	0.83 (0.26–2.62)	51 (36.4)	0.44**	0.24** (0.10–0.56)	2 (15.4)	1.11	0.30 (0.04–2.35)	10 (55.6)	2.04	1.06 (0.27–4.13)
Colo-rectal	28 (22.1)	2.05**	1.93* (1.07–3.48)	18 (19.6)	1.13	0.78 (0.41–1.49)	16 (38.1)	1.72	1.62 (0.72–3.68)	29 (48.3)	0.71	0.52 (0.23–1.17)	4 (14.8)	1.06	0.55 (0.13–2.36)	6 (23.1)	0.49	0.32 (0.09–1.08)
Gynecological	14 (24.6)	2.36*	2.33* (1.17–4.65)	17 (23.9)	1.46	1.47 (0.79–2.74)	10 (30.3)	1.21	1.16 (0.49–2.72)	16 (48.5)	0.71	0.68 (0.30–1.52)	5 (19.2)	1.45	1.45 (0.45–4.62)	6 (28.6)	0.65	0.65 (0.21–1.96)
Malignant melanoma	4 (9.8)	0.78	0.75 (0.25–2.26)	17 (37.8)	2.81**	2.51* (1.24–5.05)	5 (31.3)	1.27	1.29 (0.40–4.13)	8 (72.7)	2.02	1.68 (0.40–6.99)	0 (0)	−	−	3 (23.1)	0.49	0.31 (0.07–1.46)
Lung	15 (22.7)	2.13*	1.97 (0.97–4.01)	16 (28.6)	1.85	1.31 (0.66–2.60)	10 (40.0)	1.86	1.46 (0.57–3.72)	14 (66.7)	1.52	1.13 (0.39–3.27)	4 (50.0)	6.08*	4.02 (0.75–21.7)	7 (43.8)	1.27	0.86 (0.25–3.02)
Lymphoma	7 (21.9)	2.03	1.95 (0.76–5.00)	15 (34.1)	2.39*	2.05 (0.99–4.27)	9 (40.9)	1.93	2.19 (0.81–5.89)	8 (53.3)	0.87	0.72 (0.22–2.36)	2 (22.2)	1.74	1.65 (0.29–9.26)	4 (36.4)	0.93	0.69 (0.17–2.88)
Head and neck	9 (32.1)	3.43**	3.32** (1.34–8.22)	13 (35.1)	2.51*	2.00 (0.90–4.43)	6 (42.9)	2.09	2.09 (0.64–6.85)	7 (63.6)	1.33	1.24 (0.32–4.81)	4 (28.6)	2.43	1.63 (0.36–7.29)	6 (50.0)	1.63	0.97 (0.23–4.10)
Other	26 (16.9)	1.47	1.41 (0.77–2.57)	34 (22.7)	1.36	0.98 (0.56–1.72)	26 (33.3)	1.39	1.48 (0.72–3.04)	38 (55.9)	0.96	0.77 (0.35–1.69)	10 (25.0)	2.03	1.25 (0.39–4.05)	18 (28.6)	0.65	0.43 (0.16–1.13)

For each area (physical, emotional, family oriented, sexual, work-related, and financial) number, frequencies and crude and adjusted odds ratios (ORs) are shown with regard to sex, age, and cancer diagnosis.

^a^Adjusted for sex, age group, and cancer diagnosis

*p < 0.05

**p < 0.01

## Discussion

### Main findings

Needs for physical and psychological rehabilitation were equally frequent. One third of the cancer patients alive at 14 months post- diagnosis experienced these needs. Women were more likely to express a need for rehabilitation; they took part more often in activities relevant for psychological and physical problems and had, to a higher extent, fulfilled their emotional needs. In general, older cancer patients were less likely to express a need for and participate in rehabilitation. Unmet needs among elderly who wanted professional help were, however, more frequent compared with younger patients.

### Strengths and weaknesses

Our results are based on a large population-based, consecutively sampled cohort of patients diagnosed with an incident cancer during a 1-year period in two representative regions in Denmark. The sampling was based on administrative data. Misclassification of the cancer diagnosis is a potential limitation. However, the regional hospital’s Patient Administrative System is used for administrative purposes, and the validity is high [23]. In addition, we reduced misclassification by asking the GPs whether the cancer diagnosis was correct.

Due to incomplete use of the additional AZCA-1 code which was not known until after the sampling, 38% of potentially eligible patients were not identified by the administrative sampling procedure. However, it was random patients who did not get the code, and analyses showed only minor differences between the patients included and the full sample of all eligible patients (data not shown). The two most pronounced differences were that of the primarily included patients 19% had breast cancer compared with 16% in the full sample. This could have slightly overestimated our results regarding need for and participation in rehabilitation. Secondly, an under-sampling of the oldest patients (+80 years) was seen compared with the full sample (13% vs. 17%). This could have underestimated the absolute figures for unmet rehabilitation needs. However, as we calculated the relative associations and adjusted for differences in sex, age, and cancer type, this selection bias can be regarded as negligible.

The response rate was high among the patients who received the questionnaire. Among responders, there was a slight overrepresentation of women, the 60–69-year-olds and patients with breast cancer (Table 1). Hence, the absolute figures for needs and activities might be overestimated. This could also be caused by the fact that individuals who respond may tend to be those in need and active in relation to rehabilitation. However, as this might have made the group included in the analyses slightly more homogeneous with less variation, the relative associations found in this study may actually be underestimated.

Participants were asked about needs and activities for a 14-month period, but the cancer disease and cancer-related activities are likely to be of major importance to most patients and therefore remembered for a long time. Hence, recall bias is supposedly low.

An important question is whether our results are generalizable. In Denmark, there are relatively small regional differences with respect to organization of the health care system and prevalence of diseases. Therefore, we assume that our results are generalizable to all of Denmark. Furthermore, our findings that patient characteristics were associated with needs, participation, and unmet needs are likely to be generalizable to other countries with similar health care systems. It must be kept in mind that the results represent cancer patients who survived for more than 1 year and therefore may not apply to short-time survivors.

### Comparison with other studies

Similar to our results, a Norwegian study including the ten most frequent cancer types (*n* = 1,325) showed that need for physical rehabilitation followed by psychological rehabilitation was the most frequent [5]. They also reported that breast cancer patients were more likely to report a need for physical rehabilitation, and we showed that they are more frequent participants in physical activities. This most likely reflects a rehabilitation need in relation to physical problems such as limitation in arm mobility and lymphedema, and furthermore, that preventive physiotherapy is systematically offered at some hospitals to this patient group. Interventions with a physical component have been found to improve physical functioning, strengths, emotional wellbeing, and reduce fatigue among cancer survivors [27], and a more systematic approach may therefore benefit all cancer patients.

Our results showed that, compared with men, women, to a higher extent, expressed needs and participated in rehabilitation. This could reflect that women to a higher extent have a need for rehabilitation, more often articulate a need for help, or that current rehabilitation offers appeal more to women, i.e., activities match female demands and values. A cross-sectional study of 1,876 Danish cancer survivors participating in a 1-week residential rehabilitation course, offered to all cancer patients, support this explanation, as 85% of the participants were women [28]. In another cross-sectional study of 396 cancer patients with various diagnoses, significant gender differences were found with regard to health care preferences [29]. Furthermore, our results show that men have significantly higher emotional unmet needs, indicating that rehabilitation efforts should be gender-tailored.

Only a few smaller cross-sectional studies have assessed utilization of rehabilitation activities among cancer patients [7, 8]. As these studies mainly included patients with breast cancer or assessed utilization of one single activity, direct comparison with our study is difficult. However, utilization of activities in these studies was also significantly higher among younger age groups. Our study adds to present knowledge that younger patients in general express a greater need for rehabilitation, presumably related to multiple challenges to handle in that period of life. At the same time, it could be an indication that the health care system does not always identify the needs among elderly, for whom it may be more difficult to ask for and seek out services. We found that elderly, who had expressed rehabilitation needs, more often had them unresolved.

A Danish survey including cancer patients at various sites (*n* = 1,490) found that half of the patients who needed psychological counseling did not receive it [9]. In our study, we distinguished between three different psychological unmet needs, showing a much higher extent of sexually unmet needs compared with emotional and family oriented unmet needs. We believe that it is crucial to discriminate between different psychological needs, and our findings underline a need for health care professionals to address delicate issues, including sexual problems. Several studies of women with breast cancer have confirmed that unmet needs in both the sexual and psychological area are of relevance. [12, 14–16, 30, 31].

### Conclusion and implications

In conclusion, one third of the total group of cancer patients reported a need for physical rehabilitation and in one third, a need for psychological rehabilitation. Half of the patients participated in one or more rehabilitation activities. Unmet needs were most often reported in the psychological, sexual, and financial areas. We observed a substantial variation in these matters pertinent to disease and patient characteristics. This study suggests that cancer care ought to systematically address the wide range of needs in all groups through integration of systematic needs assessment and targeted supply of offers. Emphasis should be put on development of assessment and monitoring tools for use in everyday clinical practice.
